# A functional conserved intronic G run in HIV-1 intron 3 is critical to counteract APOBEC3G-mediated host restriction

**DOI:** 10.1186/s12977-014-0072-1

**Published:** 2014-08-29

**Authors:** Marek Widera, Frank Hillebrand, Steffen Erkelenz, Ananda Ayyappan Jaguva Vasudevan, Carsten Münk, Heiner Schaal

**Affiliations:** Institute for Virology, Medical Faculty, Heinrich-Heine-University Düsseldorf, Universitätsstraße 1, Düsseldorf, 40225 Germany; Clinic for Gastroenterology, Hepatology, and Infectiology, Medical Faculty, Heinrich-Heine-University Düsseldorf, Moorenstr. 5, Düsseldorf, 40225 Germany; Current address: Institute of Virology, University Hospital of Essen, University Duisburg-Essen, Virchowstr. 179, Essen, 45147 Germany

**Keywords:** HIV-1 infection, Host restriction, Cytidine deaminase, APOBEC3G, Viral infectivity factor (Vif), Viral protein R (Vpr), Alternative pre-mRNA splicing, G run, hnRNP F/H, Locked nucleic acids (LNAs)

## Abstract

**Background:**

The HIV-1 accessory proteins, Viral Infectivity Factor (Vif) and the pleiotropic Viral Protein R (Vpr) are important for efficient virus replication. While in non-permissive cells an appropriate amount of Vif is critical to counteract APOBEC3G-mediated host restriction, the Vpr-induced G2 arrest sets the stage for highest transcriptional activity of the HIV-1 long terminal repeat.

Both *vif* and *vpr* mRNAs harbor their translational start codons within the intron bordering the non-coding leader exons 2 and 3, respectively. Intron retention relies on functional cross-exon interactions between splice sites A1 and D2 (for *vif* mRNA) and A2 and D3 (for *vpr* mRNA). More precisely, prior to the catalytic step of splicing, which would lead to inclusion of the non-coding leader exons, binding of U1 snRNP to the 5*'* splice site (5*'*ss) facilitates recognition of the 3*'*ss by U2 snRNP and also supports formation of *vif* and *vpr* mRNA.

**Results:**

We identified a G run localized deep in the *vpr* AUG containing intron 3 (G_I3_-2), which was critical for balanced splicing of both *vif* and *vpr* non-coding leader exons. Inactivation of G_I3_-2 resulted in excessive exon 3 splicing as well as exon-definition mediated *vpr* mRNA formation. However, in an apparently mutually exclusive manner this was incompatible with recognition of upstream exon 2 and *vif* mRNA processing. As a consequence, inactivation of G_I3_-2 led to accumulation of Vpr protein with a concomitant reduction in Vif protein. We further demonstrate that preventing hnRNP binding to intron 3 by G_I3_-2 mutation diminished levels of *vif* mRNA. In APOBEC3G-expressing but not in APOBEC3G-deficient T cell lines, mutation of G_I3_-2 led to a considerable replication defect. Moreover, in HIV-1 isolates carrying an inactivating mutation in G_I3_-2, we identified an adjacent G-rich sequence (G_I3_-1), which was able to substitute for the inactivated G_I3_-2.

**Conclusions:**

The functionally conserved intronic G run in HIV-1 intron 3 plays a major role in the apparently mutually exclusive exon selection of *vif* and *vpr* leader exons and hence in *vif* and *vpr* mRNA formation. The competition between these exons determines the ability to evade APOBEC3G-mediated antiviral effects due to optimal *vif* expression.

**Electronic supplementary material:**

The online version of this article (doi:10.1186/s12977-014-0072-1) contains supplementary material, which is available to authorized users.

## Background

The *Human immunodeficiency virus type 1* (HIV-1) exploits cellular components of the host cell for efficient replication, while being counteracted by so called host restriction factors, which have antiviral properties and negatively affect viral replication.

Currently known host restriction factors consist of five major classes that are the DNA deaminase subfamily APOBEC3 (apolipoprotein B mRNA-editing enzyme, catalytic polypeptide-like) [1–3], the Ubl conjugation ligase TRIM5α (Tripartite motif-containing protein 5 alpha) [4–6], the integral membrane protein BST-2 (bone stromal tumor protein 2)/tetherin [7,8], the dNTP hydrolase and RNase SAMHD1 (SAM domain and HD domain-containing protein 1) [9–13], and the tRNA binding protein SLFN11 (Schlafen 11) [14–16]. The APOBEC3 (A3) family includes seven members (A3A to A3D and A3F to A3H) that are located in a gene cluster on chromosome 22 [17–19], from which A3D, A3F, A3G and A3H have HIV-1 restrictive capacities [20–22]. They are encapsidated in newly assembled virions, and following the subsequent infection of a host cell, introduce C-to-U substitutions during minus-strand synthesis. This results in G-to-A hypermutations in the HIV-1 genome, which negatively impact viral replication. Hereby, A3G causes *G*G to *A*G transitions, whereas A3D, A3F, and A3H lead to an overrepresentation of *G*A to *A*A hypermutations [20,21,23–26]. However, the HIV-1 encoded accessory protein Vif counteracts the four A3 proteins by binding CBFβ and recruiting an E3 ubiquitin ligase complex, thus inducing their polyubiquitination and proteasomal degradation [20,27].

Since all early HIV-1 proteins are expressed from spliced intronless viral mRNAs, splicing factors and splicing regulatory proteins are particularly involved in viral infection. Moreover, CAP-dependent translation is initiated by binding of the 40S ribosomal subunit at the mRNAs’ 5′end and by ribosomal scanning for an efficient AUG. By using at least four 5′ splice sites (5′ss) and eight 3′ splice sites (3′ss), the HIV-1 9 kb pre-mRNA is processed into more than 40 alternatively spliced mRNA isoforms [28] encoding at least 18 HIV-1 proteins, most of them interacting with a wide variety of host cell components [29]. Thus, HIV-1 relies on massive alternative splicing to bring each of its eight translational start codons (*gag*-*pol*, *vif*, *vpr*, *tat*, *rev*, *nef*, *vpu*, and *env*) into close proximity of the 5′cap of the respective alternatively spliced mRNA. The only exception to this rule is the *env* ORF, which is translated from the bicistronic *vpu/env* mRNA. Here, a minimal upstream ORF upstream of the *vpu* ORF allows efficient translation initiation at the downstream *env* AUG [30,31].

**Figure 1:**
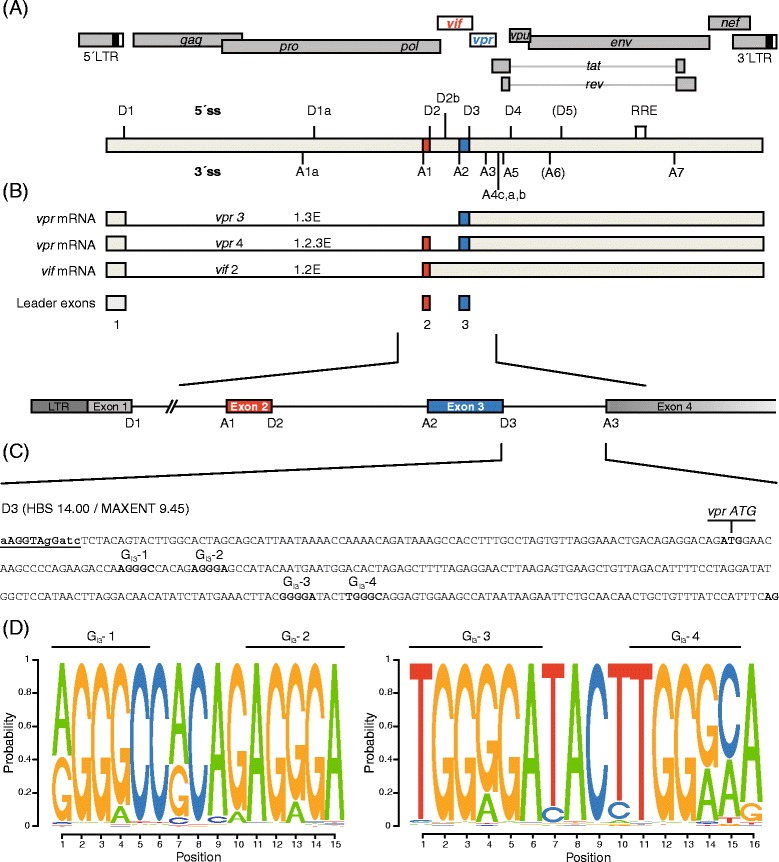
**Schematic drawing of the HIV-1 NL4-3 genome. (A)** The diagram illustrates the HIV-1 provirus genome including locations of open reading frames (ORFs), long terminal repeats (LTRs), 5′ and 3′ splice sites (ss), exons, introns, and the Rev response element (RRE). *Vif* and *vpr* exons and ORFs are highlighted in red and blue, respectively. The RRE is indicated by an open box. **(B)** *Vif* and *vpr* mRNA are formed primarily by splicing of 5′ss D1 to 3′ss A1 and A2, respectively. The noncoding leader exons 2 and 3 are included and AUG-containing introns are retained. **(C)** Sequence of intron 3 in the proviral HIV-1 genome including the locations of the *vpr* AUG and G runs G_I3_-1 to G_I3_-4. The intrinsic strength of 5′ss D3 is indicated (HBS, MAXENT). **(D)** Sequence logos generated from a sequence alignment of the HXB2 regions from nt 5581 - nt 5597 and nt 5708 - nt 5723, respectively, flanking the four G runs G_I3_-1 to G_I3_-4. Sequences were obtained from the Los Alamos HIV Sequence Database (http://www.hiv.lanl.gov). The relative size of the letters reflects the relative frequency of the nucleotides at the position in the alignment.

Within the 4 kb class of mRNAs (Figure 1A-B), downstream of 5′ss D2–D4 translational start codons are localized, which can only be recognized by the 40S ribosomal subunit if the respective introns are retained. In particular, *vif* mRNA is formed when the intron upstream of exon 2 is spliced out, while its downstream intron is retained. In a similar way, *vpr* mRNA is formed by removing upstream introns carrying translational inhibitory AUGs but repressing D3 and thus retaining intron 3. Both mRNAs rely on functional cross-exon interactions between the 5′ss and the corresponding upstream 3′ss [32–34]. Thus, formation of unproductive spliceosomal complexes at the 5′ss is essential for 3′ss activation and exon definition as well as for splicing-repression at the 5′ss [35]. Hence, the expression levels of *vif* and *vpr* mRNAs are dependent on U1 bound, but splicing repressed 5′ss [32,33].

Notably, excessive splicing at A2 was shown to result in detrimental impairment of the balanced ratio of spliced to unspliced viral mRNAs and loss of the viral unspliced genomic 9 kb mRNA, a phenotype referred to as oversplicing [36,37]. Since Gag and Pol are encoded by the unspliced 9 kb mRNA, oversplicing decreases the amounts of all Gag and Pol proteins including p55Gag and p24-CA resulting in massive inhibition of viral particle production and replication [36–39].

Moreover, transcripts containing either non-coding leader exon 2 or 3 as required for *vif* and *vpr* mRNAs, respectively, appear to be regulated in a similar way as 3′ss A1 and A2 recognition, which appears to underlie a mutually exclusive selection [33]. However, the molecular mechanism is still poorly understood.

Since 3′ss A2 was shown to be an intrinsically strong 3′ss [40], *trans*-acting elements are necessary to repress its usage. Indeed, the ESSV within the non-coding leader exon 3, which consists of three UAG motifs, has been reported to inhibit splicing at 3′ss A2 [41–43]. In addition, the Tra2-alpha and Tra2-beta-dependent splicing regulatory element ESE_*vpr*_ positively regulates balanced amounts of exon 3 recognition by acting positively on U1 snRNP recruitment to 5′ss D3, which in turn promotes recognition of the upstream 3′ss A2 via cross exon interaction [33]. Vpr formation was further proposed to be regulated by high-mobility group A protein 1a (HMGA1a), which binds immediately upstream of 5′ss D3 and acts to repress splicing at this position. Here, trapping of U1 snRNP might activate 3′ss A2 and repress splicing at 5′ss D3 [44].

Recently, we identified a G run with high affinity for hnRNP F/H and A2/B1 proteins localized within intron 2 (G_I2_-1), but upstream of the *vif* AUG, which represses usage of the alternative 5′ss D2b [34]. Mutations of G_I2_-1 led to considerable upregulation of *vif* mRNA expression [34]. Here, we analyzed whether regulation of exon 3 inclusion and processing of *vpr* mRNAs is regulated in an analogous manner by intronic G runs located in HIV-1 intron 3.

## Results

### The guanosine run element (G_I3_-2) localized deeply within HIV-1 intron 3 is critical for efficient replication in PBMCs

**Figure 2:**
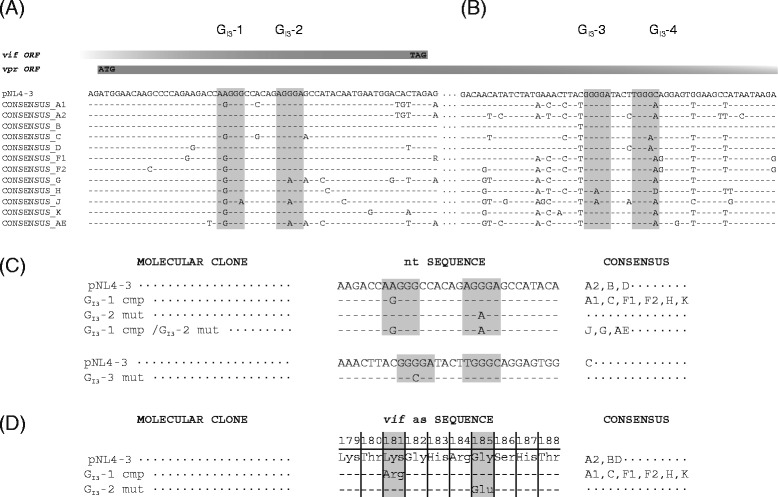
**Comparison of intron 3 G runs and their sequence surroundings in HIV-1 subtypes. (A-B)** Proviral DNA sequence surroundings of the HIV-1 consensus sequences A1 to AE of G_I3_-1 to G_I3_-4. Conserved sequences are represented by –, deviants by letters. Conserved G run motifs are highlighted by grey boxes. The ORF of *vif* and *vpr* including start and stop codons are indicated as declining boxes. The subtype sequences were analyzed with the RIP 3.0 software (http://www.hiv.lanl.gov/content/sequence/RIP/RIP.html). **(C)** Molecular clones of pNL4-3 used in this study. Sequences of G runs G_I3_-1 and G_I3_-2 including surrounding nucleotides are depicted. Mutated sequences are represented by letters. Corresponding consensus sequences are indicated on the right. **(D)** The amino acid substitutions of the proviral clones used in this study. The sequence of G runs G_I3_-1 and G_I3_-2 including surrounding nucleotides is depicted. Substituted amino acids and their position in Vif Protein are shown in the table. Corresponding consensus sequences are indicated on the right.

**Figure 3:**
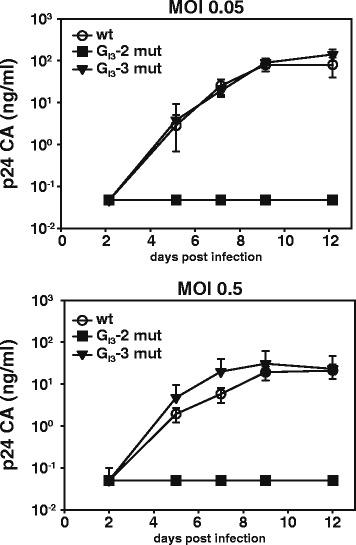
**G run G**_**I3**_ **-2 is crucial for efficient virus replication in PBMCs.** Peripheral blood mononuclear cells from two healthy donors were infected with NL4-3 virus or mutant derivatives with the indicated multiplicity of infection (MOI). Virus production was determined by p24-CA capture ELISA of cell-free supernatant collected at the indicated time points.

Previously we have shown that an intronic G run within HIV-1 intron 2 is critical for splicing regulation of *vif* mRNA [34]. To examine whether an intronic G run is likewise critical for regulation of *vpr* mRNA, whose processing similarly depends on intron retention, we inspected HIV-1 intron 3 for the occurrence of G runs. Since they are highly abundant in mammalian introns [45–47], it was not surprising that we found four G runs, which we termed G_I3_-1 to G_I3_-4 according to their 5′ to 3′ localization (Figure 1). However, only two of these, G_I3_-2 and G_I3_-3, were found to match the consensus motif DGGGD (where D is G, A, or T) of the high affinity binding site for members of the hnRNP F/H family [48]. Moreover, since G_I3_-2 and G_I3_-3 were highly conserved in HIV-1 strains (Figure 1D), we analyzed whether one or even both had an impact on viral replication. To this end, we disrupted each of them in the molecular clone pNL4-3 by introducing single nucleotide substitutions (pNL4-3 G_I3_-2 mut: AGGGA > AGAGA, pNL4-3 G_I3_-3 mut: GGGGA > GGCGA). Since for G_I3_-2 it was not possible to introduce a mutation without changing the coding sequence of the overlapping *vif* and *vpr* open reading frames (ORFs), we chose a nucleotide substitution, which was present in HIV-1 subtypes J, G, and AE (Figure 2A, C). This exchange, however, resulted in a single amino acid substitution within the C terminus of Vif (AGGGA > AGAGA, G185E). To be able to infect PBMCs with equal amounts of viral particles, we first transfected HEK 293 T cells with the proviral plasmid pNL4-3 or its mutant derivates, pNL4-3 G_I3_-2 mut or pNL4-3 G_I3_-3 mut, and then harvested virus-containing supernatants 48 h post transfection. The TCID_50_ were calculated by X-Gal staining of infected TZM-bl reporter cells. These cells carry a luciferase and β-galactosidase expression cassette under the control of the HIV-1 LTR and thus express both reporter genes in the presence of HIV-1 Tat [49]. With a multiplicity of infection (MOI) of each of 0.05 and 0.5, PBMCs from two healthy donors were then infected and p24-CA protein levels were determined at various time points. As shown in Figure 3, G_I3_-3, but not G_I3_-2 mutated virus, was able to replicate in PBMCs indicating that specifically G_I3_-2 was critical for efficient virus replication in primary T cells.

### Mutating G_I3_-2 results in an impaired ratio of spliced to unspliced mRNAs

**Figure 4:**
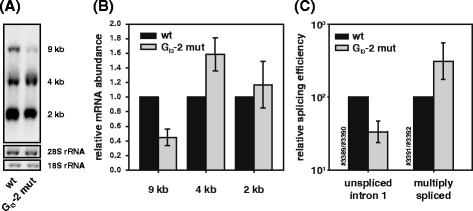
**G**_**I3**_ **-2 causes alterations in mRNA processing. (A)** Northern blot analysis of total RNA isolated from HEK 293 T cells transfected with wild type or mutant pNL4-3 was isolated 48 h post transfection. RNA was separated on a 1% RNA agarose gel, capillary blotted, and cross-linked on a positively charged nylon membrane and UV cross-linked. The membrane was treated with a DIG-labelled DNA fragment binding to exon 7. **(B)** Quantitation of relative amounts of mRNAs from (A). **(C)** RNA from panel A was subjected to quantitative RT-PCR analysis using a primer pair specific for intron 1 containing mRNAs of the 9 kb mRNA class (#3389/#3390), and intronless mRNAs of the 2 kb class (#3391/#3392), which were normalized to exon 7 containing mRNAs (#3387/#3388).

In order to investigate the molecular cause for the replication defect of G_I3_-2 mutant virus, we analyzed the splicing patterns of proviral DNA from pNL4-3 and G run mutant. To this end, total RNA of HEK 293 T cells transfected with each of the proviral DNAs was subjected to Northern blot analysis and probed with a DIG-labeled HIV-1 exon 7 amplicon detecting all viral mRNA classes. While the overall splicing pattern was not changed for the G_I3_-3 provirus (data not shown), inactivation of G_I3_-2 caused massive disturbance of the balanced ratio of the three viral mRNA classes with the most obvious decrease in the amount of unspliced 9 kb mRNA (Figure 4A-B). Concomitantly, the ratio of 2 and 4 kb mRNA classes was increased indicating massive splicing defects (Figure 4B).

In order to quantify the amounts of the viral RNA classes, we performed quantitative RT-PCR analysis using primers (Additional file 1: Figure S1) binding in intron 1 (*gag*-*pol*) to detect unspliced 9 kb mRNA, as well as primers to quantify the relative amount of multiply spliced mRNAs (exon junction D4/A7). As shown in Figure 4C, the relative amount of unspliced, i.e. intron 1 containing mRNAs, was three-fold decreased compared to the amount from non-mutated virus. In parallel, the relative amount of multiply spliced mRNAs was three-fold increased. Thus, inactivation of G_I3_-2 shifted the balance towards intronless viral mRNAs.

Since p24-CA protein is encoded by the unspliced 9 kb mRNA, the widening gap between unspliced and multiply spliced mRNAs that has been previously described and referred to as oversplicing or excessive splicing [36–39] might result in diminished viral p24-CA production. However, since unspliced 9 kb mRNA was still detectable in the Northern blot analysis of G_I3_-2 mutant virus, a lower level of viral particle production was probably not the only cause of the totally abolished replication of G_I3_-2 mutant virus in PBMCs.

### G_I3_-2 plays a major role in exon 2 vs. exon 3 selection and *vif* vs. *vpr* mRNA expression

**Figure 5:**
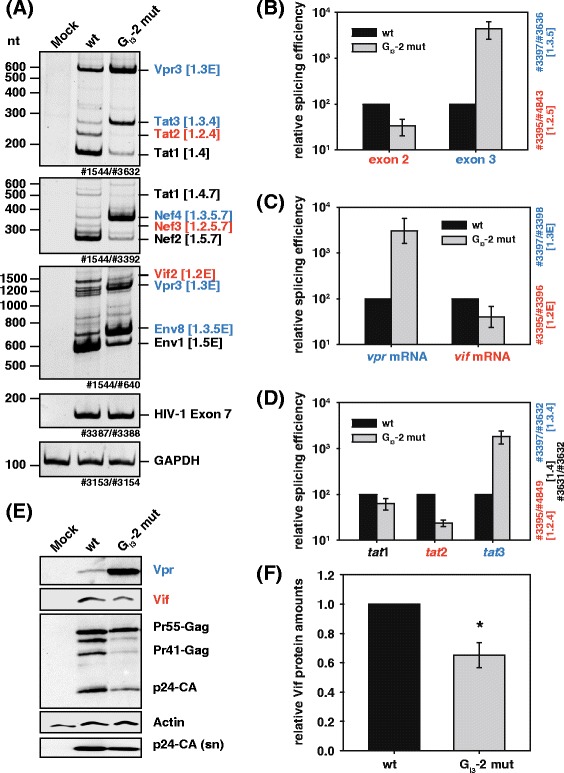
**Mutation G**_**I3**_ **-2 increases** ***vpr*** **, but decreases** ***vif*** **mRNA and Vif protein levels. (A)** RT-PCR analysis of RNA from HEK 293 T cells transiently transfected with pNL4-3 or its G_I3_-2 mutant derivate. Compare with Additional file 1: Figure S1 for specific primer binding sites. RNA was isolated 48 h post transfection. Primer pairs are indicated at the bottom of each panel, transcript isoforms on the right. To compare total RNA amounts, separate RT-PCRs were performed by using primer pairs amplifying HIV-1 exon 7 and cellular GAPDH sequence. PCR amplicons were separated on a non-denaturing polyacrylamide gel (10%) and stained with ethidium bromide. **(B-D)** Quantitative RT-PCR of total RNA from (A) using primers indicated in Additional file 1: Figure S1. The NL4-3 splicing pattern (wt) was set to 100% and the relative splice site usage was normalized to exon 7 containing HIV-1 transcripts. **(E)** Immunoblot analysis of the indicated proteins employing lysates or pelleted virions from supernatant (sn) obtained from HEK 293 T cells that were transiently transfected with wild type or G_I3_-2 mutant proviral DNA. Transfected cells were lysed in RIPA buffer and the lysates were collected 48 h post transfection. Cell-free supernatant was concentrated by sucrose centrifugation. **(F)** Quantification of Vif protein amounts from **(E)**.

Since activated PBMCs exhibit high expression of the host restriction factor A3G [34,50], we were interested in whether the replication defect of G_I3_-2 mutant virus might have originated from disturbed expression of the viral antagonist of A3G, which is the accessory protein Vif. For this purpose, we analyzed the impact of the G_I3_-2 inactivating mutation on *vif* gene expression. HEK 293 T cells were transiently transfected with pNL4-3 or the G_I3_-2 mutant proviral plasmid pNL4-3 G_I3_-2 mut, and total RNA and proteins were harvested 48 h post transfection. As determined by semi-quantitative RT-PCR using primer pairs to specifically amplify intron-containing (4 kb) or intronless (2 kb) HIV-1 mRNAs (Additional file 1: Figure S1), inactivation of G_I3_-2 resulted in excessive exon 3 splicing in the *tat*, *nef*, and *env* mRNAs (Tat3, Nef4, Env8), and concomitantly led to accumulation of *vpr* mRNA indicating that G_I3_-2 represses exon 3 and 3′ss A2 recognition (Figure 5A). However, enhanced splicing of A2 was obviously incompatible with the recognition of the upstream exon 2 as observed by means of multiply spliced mRNAs (Tat2, Nef3) and consequently *vif* mRNA processing (Figure 5A). Mutating G_I3_-2 considerably shifted from exon 2 to exon 3 containing transcripts indicating that G_I3_-2 balances selection of exon 2 and exon 3.

To quantify the impact of inactivated G_I3_-2 on balanced regulation of exon 2 and 3 splicing, we performed quantitative real time RT-PCR using primer pairs (c.f. Additional file 1: Figure S1 for primer binding sites) detecting the relative splicing efficiencies of mRNAs containing either exon 2 or 3 as well as the relative splicing efficiencies of *vpr* and *vif* mRNAs (Figure 5B and C). We quantified a 44-fold increase in exon 3 and concomitant three-fold decrease in exon 2 containing transcripts (Figure 5B). Furthermore, we quantified a 30-fold increase of *vpr* mRNA, when G_I3_-2 was mutated confirming that G_I3_-2 is also required for the repression of 3′ss A2 (Figure 5C). On the other hand, *vif* mRNA was observed to decrease 2.5-fold compared to the non-mutated virus, verifying the aforementioned observation that 3′ss A1 and A2 are spliced in an apparently mutually exclusive manner (Figure 5C). This was furthermore confirmed by the quantitation of the relative splicing efficiency of *tat* mRNAs of G_I3_-2 mutant virus, which resulted in considerable increase in *tat3* (18-fold) and concomitant decrease of *tat2* (four-fold) mRNA splicing (Figure 5D).

Next, we performed Western blot analyses to evaluate excessive exon 3 splicing and opposite effects on *vpr* and *vif* mRNA splicing also on protein levels (Figure 5E and F). In accordance with decreased amounts of unspliced mRNAs, we observed a remarkable decrease in Gag expression, which was mainly reflected by the reduced amounts of its cleavage products. Similarly, virus particles in the supernatant were decreased (Figure 5E, p24-CA (sn)). As expected from the RT-PCR results described above, the expression of Vpr protein was considerably increased when G_I3_-2 was mutated. In parallel, Vif protein amounts were significantly decreased to 65% when compared to non-mutated virus (Figure 5E and F). In conclusion, the intronic G run G_I3_-2 acts to repress the activation of 3′ss A2 and plays a major role in the apparently mutually exclusive selection of exon 2 and exon 3, which in turn regulates the expression of Vpr and Vif protein.

### G_I3_-2 is critical for viral replication in APOBEC3G-expressing but not -deficient cells

**Figure 6:**
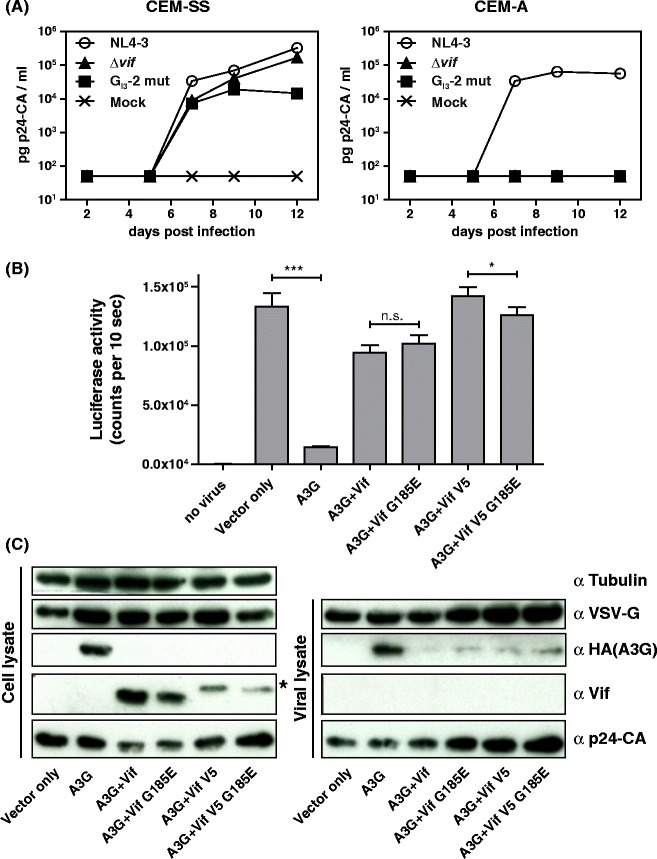
**G**_**I3**_ **-2 is critical for efficient virus replication in A3G expressing cells. (A)** CEM-SS and CEM-A cells were infected with wild type or G_I3_-2 mutant NL4-3 virus and virus production was determined by p24 CA capture ELISA of cell-free supernatant collected at the indicated time points. **(B)** Vif G185E mutant counteracts A3G with comparable efficiency as wild type Vif. Viral rescuing activity of Vif and Vif G185E mutant against A3G compared to vector only control (no A3G). HIV-1 Δvif.luc reporter viruses were generated in the presence of A3G and absence of A3G (vector control), and A3G in combination with Vif, Vif G185E, Vif-V5, and Vif-V5 G185E plasmids. Virions were normalized by RT activity. Luciferase activity was measured 48 hours post infection. Unpaired *t* tests were computed to determine the differences between the group of samples reached the level of statistical significance (ns, not significant; *, *P* 0.05 and ***, *P* 0.0001). **(C)** Viral supernatants were concentrated through a 20% sucrose cushion by ultracentrifugation. Immunoblot analysis of the expression and encapsidation of A3G in the presence and absence of Vif, Vif G185E, Vif-V5, and Vif-V5 G185E: A3G was detected by an anti (α)-HA antibody. Vif, VSV-G, and p24-CA were detected by their respective monoclonal antibodies. Tubulin served as loading control for cell lysates and p24-CA and VSV-G for viral lysates. Asterisk in the Vif blot indicates the shift in the molecular weight of V5-tagged Vif.

Since physiological levels of Vif are necessary to counteract A3G-mediated host restriction, we were interested in whether the diminished Vif protein levels of G_I3_-2 mutated virus were the underlying cause of the replication incompetence in PBMCs. In order to prove this hypothesis, we aimed to analyze the replication kinetics of mutant and non-mutant virus in A3G low expressing CEM-SS [2,51–53] and high expressing CEM-A [54] cell lines, whose expression we previously confirmed by A3G immunoblot analysis [34]. As a control, the *vif* deficient NL4-3 *Δvif* virus was included in this analysis [55]. CEM cells were infected with an MOI of 0.01, cell free supernatants were harvested at frequent intervals, and p24 capsid protein production (CA) was monitored by capture ELISA to quantify viral replication (Figure 6A). As anticipated, in A3G low expressing CEM-SS cells, *vif* deficient virus was able to produce viral particles with comparable efficiency as non-mutant NL4-3 virus. However, the replication curve of G_I3_-2 mutant virus flattened out at a tenfold lower p24-CA amount compared to non-mutant and *vif* deficient virus, confirming that inactivating G_I3_-2 not exclusively alters *vif* mRNA processing but generally disturbs the balanced ratio of all classes of RNA impairing viral replication. On the contrary, *vif* deficient as well as G_I3_-2 inactivated viruses were replication incompetent in A3G high expressing CEM-A cells and thus ended up in an abortive infection. These results indicate that the replication incompetence was the net result of diminished Vif protein levels as well as reduced virus production caused by the G run mutation. These results also demonstrate that G_I3_-2 was critical for efficient virus replication in A3G expressing cells and that the threshold of Vif required for optimal viral replication was in a narrow range.

To determine the viral rescuing activity of Vif and Vif point mutant G185E we used two different constructs that have Vif without a tag and Vif with a C-terminal V5 tag. The point mutation yielding to the amino acid substitution G185E was introduced in both plasmids by site directed mutagenesis and used to produce viral particles by transfection of HEK 293 T cells. Normalized amounts of particles were used to transduce HEK 293 T cells and the intracellular luciferase activity was quantified two days post transduction. A3G reduced the infectivity of HIV-1 luc reporter vectors about 10-fold, and the presence of Vif and Vif G185E (both versions) rescued the reporter virus infectivity to above 70% attained by HIV-1 vectors generated without A3G (Figure 6B). Immunoblot analysis of cellular and viral lysates confirmed wild type Vif- and G185E mutant Vif-triggered proteosomal degradation of A3G in the viral producer cells (Figure 6C). In the absence of Vif or Vif G185E, A3G was efficiently incorporated in the viral particles. However, in the presence of Vif or Vif G185E only traces of A3G were detectable in encapsidated viruses. The amount of Vif and Vif G185E within the viral particles was under the detection limit.

In conclusion, G_I3_-2 is critical for efficient virus replication in A3G expressing cells while G185E mutation does not alter the Vif protein’s counteracting function.

### Inhibition of hnRNP protein binding to the intronic G run G_I3_-2 restricts viral particle production

**Figure 7:**
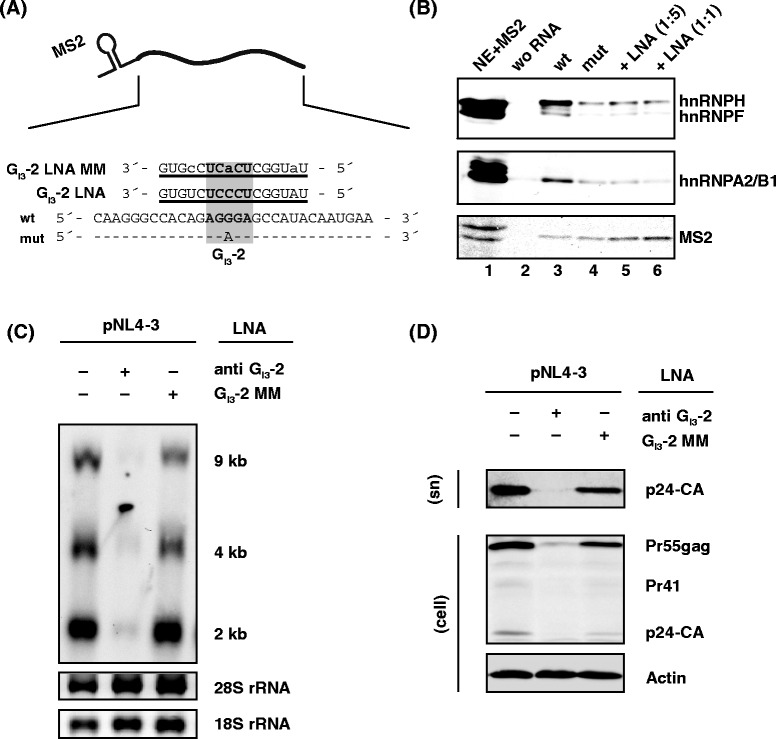
**G**_**I3**_ **-2 is specifically bound by hnRNP F/H and A2/B1. (A)** Schematic illustration of the RNA pull-down experiment and binding site of locked nucleic acids directed against G_I3_-2 within HIV-1 intron 3. **(B)** RNA pull-down assay using HeLa nuclear extract. Substrate RNAs containing a MS2 sequence and the wild type or mutant G runs sequence were covalently linked to adipic acid dihydrazide-agarose beads and incubated with HeLa cell nuclear protein extract. MS2-proteins were added to monitor the RNA input. For interference with protein:RNA interaction, wt RNA was pre-incubated with the G_I3_-2 LNA in a ratio of either 1:5 or 1:1 relative to the amount of RNA substrate. The precipitated proteins were resolved by SDS-PAGE (16%) and detected by immunoblot analysis using anti hnRNP F/H and A2/B1 antibodies. MS2 specific antibodies were used as a loading control. **(C)** HeLa cells were co-transfected with pNL4-3 and locked nucleic acids (LNAs) masking G_I3_-2 or the respective mismatch control. Total RNA was isolated 24 h post transfection and subjected to Northern blotting using a HIV-1 specific probe. **(D)** Immunoblot analysis of p24-CA using cellular lysates (cell) and pelleted virions from the supernatant (sn) of co-transfected cells from (C).

Since G runs were demonstrated to act as high affinity binding sites for members of the hnRNP F/H and A2/B1 protein families [34,48], RNA affinity precipitation assays were performed to screen for potential interaction also with the viral G run G_I3_-2. Therefore, short RNA oligonucleotides containing an MS2 coat protein RNA stem loop (Figure 7A), and either the wild type or mutant G_I3_-2 sequence, were transcribed *in vitro*. The RNAs were then covalently immobilized on agarose beads and incubated in HeLa cell nuclear extract supplemented with recombinant MS2 coat protein to allow monitoring RNA pull-down efficiency. Subsequently, the associated proteins were eluted and separated on SDS-PAGE and subjected to immunoblot analysis (Figure 7B). As expected, we could detect high levels of hnRNP F/H and A2/B1 proteins on RNAs containing the wild type G_I3_-2 sequence, while these were markedly reduced for the mutated RNA substrate (Figure 7B, upper and middle panel, lanes 3 and 4). Noticeably, equal levels of MS2 protein detected on wild type and mutated RNAs indicated comparable precipitation efficiencies for both RNAs (Figure 7B, lower panel, lanes 3 and 4) and therefore suggested that hnRNP F/H and/or A2/B1 may act through the G_I3_-2 sequence to contribute to splicing regulation of *vif* and *vpr* mRNAs.

To test whether hnRNP binding can be prevented by masking G_I3_-2 sequence using an RNA-based antisense approach, we also determined the precipitated levels of hnRNP F/H and A2/B1 proteins together with wild type RNAs in presence of a locked nucleic acid (LNA) specifically targeting the G_I3_-2 sequence (G_I3_-2 LNA). Herein, decreased levels of hnRNP F/H and A2/B1 proteins were detected on wild type RNA to which the G_I3_-2 LNA had been added (Figure 7B, cf. lanes 3 and 5). Moreover, upon increasing the concentration of the G_I3_-2 LNA, hnRNP F/H and A2/B1 precipitation efficiency could be further slightly reduced (Figure 7B, cf. lanes 3, 5 and 6). Altogether, these results demonstrate that this LNA rendered G_I3_-2 inaccessible for hnRNP F/H and A2/B1 binding.

Next, we were interested in whether the G_I3_-2 LNA might be suitable to impair viral particle production. Following co-transfection of HeLa cells with pNL4-3 and either the G_I3_-2 LNA or a control LNA (G_I3_-2 MM LNA) that contained three mismatches, total RNA was harvested and analyzed by Northern blotting using a HIV-1 exon 7 specific probe. Co-transfection of the G_I3_-2 LNA resulted in a considerable reduction in viral RNAs compared to pNL4-3 alone or pNL4-3 co-transfected with the mismatch control LNA, G_I3_-2 MM (Figure 7C). To further determine whether viral particle production and Gag protein expression were also affected, total proteins of the transfected cells and the virus containing supernatant were subjected to immunoblot analysis and detected with a p24-CA specific antibody. In line with the above findings, Gag precursor (Pr55gag) as well as Gag processing intermediate (Pr41) and product (p24-CA) were significantly reduced in the presence of the G_I3_-2 LNA (Figure 7D). There was little effect on the Gag protein expression and virus production when co-transfecting the G_I3_-2 MM LNA emphasizing the specificity of the G_I3_-2 LNA and the impact of G_I3_-2 on viral particle production.

### The G_I3_-2 binding site is functionally conserved in HIV-1

**Figure 8:**
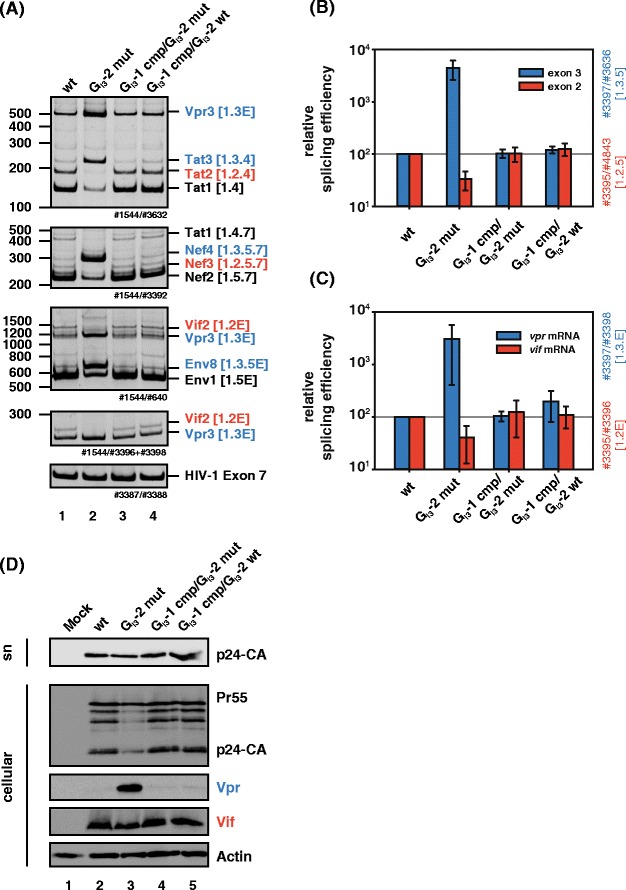
**A single G run is sufficient to maintain the HIV-1 splicing pattern. (A)** RT-PCR analysis of RNA isolated from HEK 293 T cells transiently transfected with pNL4-3 or its mutant derivates 48 h post transfection. The used primer pairs are illustrated in Additional file 1: Figure S1. Transcript isoforms are indicated on the right. Separate RT-PCRs were performed by using primer pairs amplifying HIV-1 exon 7 to compare total RNA amounts. PCR amplicons were separated on a non-denaturing polyacrylamide gel (10%) and stained with ethidium bromide. **(B-C)** Quantitative RT-PCR of total RNA obtained from panel **(A)**. The NL4-3 splicing pattern (wt) was set to 100% and the relative splice site usage was normalized to exon 7 containing HIV-1 transcripts. Compare with Additional file 1: Figure S1 for specific primer binding sites. **(D)** Immunoblot analysis of the indicated proteins employing lysates from HEK 293 T cells (cellular) and their supernatants (sn) transiently transfected with the indicated proviral DNAs. Transfected cells were lysed in RIPA buffer and lysates were collected 48 h post transfection. Virions were pelleted by sucrose centrifugation.

To assess whether G_I3_-2 might be a valuable target for LNA-mediated antiviral therapy, we were interested in whether G_I3_-2 is conserved in all HIV-1 subtypes. Indeed, an alignment of all HIV-1 consensus sequences showed that 9 out of 12 consensus sequences encode a conserved G run at the designated position (Figure 2A). The remaining three subtypes lacking a G run at this particular position contain a G run only 6 nucleotides upstream due to a compensatory nucleotide substitution in position 2 of G_I3_-1 restoring the protein binding consensus sequence DGGGD. The A > G substitution in G_I3_-1 likely converts a low affinity (AAGGGC) into a high affinity binding site (AGGGGC). To demonstrate that the compensatory G_I3_-1 mutation could functionally substitute for an inactivated downstream G_I3_-2 binding site we inserted the corresponding mutations into pNL4-3 and pNL4-3 G_I3_-2 mut (Figure 2C) and determined their splicing outcomes. Total RNA was isolated 24 h following transient transfection of HEK 293 T cells, and splicing patterns were analyzed by qualitative (Figure 8A) and quantitative RT-PCR (Figure 8B). Introducing an A > G mutation in position 2 of G_I3_-1 while G_I3_-2 was inactivated by the G > A mutation, we could compensate the excessive exon 3 and *vpr* mRNA splicing phenotype described above and restored the amounts of exon 2 containing transcripts (Figure 8A, lane 3) as well as *vif* mRNA (Figure 8B). These results demonstrate that the A > G nucleotide change in position 2 of G_I3_-1 (cf. Figure 2C; J, G, AE) is a compensatory mutation. The introduction of this substitution without inactivating downstream G_I3_-2 had no effect on *vif* and *vpr* mRNA amounts (Figure 8A-B, cf. lanes 1 and 5) suggesting that there is no evolutionary pressure on two functional binding sites. To determine whether the compensatory mutant was also capable of restoring and rescuing Vif and Vpr protein levels, we isolated total cellular proteins and subjected them to immunoblot analysis. Consistent with the findings above, the compensatory A > G mutation reduced Vpr amounts and restored Vif protein levels to those levels obtained with wild type pNL4-3 (Figure 8D, Vpr, Vif). In addition, the reduced amount in Gag precursor as well as viral particle production could be rescued (Figure 8D, p24-CA). The data obtained in these experiments highlights a functional conservation of the G run in all HIV-1 subtypes supporting an indispensable role for G_I3_-2 in HIV-1 replication.

## Discussion

Within HIV-1 NL4-3 intron 3 we identified a high affinity binding site for members of the hnRNP F/H family, termed G_I3_-2. Binding of hnRNP F/H and A2/B1 proteins to G_I3_-2 was confirmed by RNA pull-down experiments and could be efficiently prevented either by point mutation or upon co-transfection with an LNA specifically targeting G_I3_-2. Inactivation of G_I3_-2 led to aberrant alternative splicing and to a replication defective phenotype in PBMCs and A3G expressing CEM-A cells.

The G_I3_-2 inactivating mutation resulted at the same time in an amino acid (aa) substitution at position 185 (G185E) in the Vif protein, which is localized in the Gag, p7-NC, and membrane binding domain [27]. Residues 172 to 192 were shown to be involved in membrane association [56], and mutating aa positions 179 to 184 (KTKGHR > ATAGHA) resulted in 25% loss of membrane binding and decreased Pr55Gag binding [57]. However, a T > A substitution at aa position 188 of Vif had no effect on the ability to decrease A3G levels [58]. Moreover, since the G185E substitution in Vif is also present in three (G, J and AE) of twelve HIV-1 consensus sequences, we assumed that it is highly unlikely that it affects Vif’s A3G counteracting activity. However, to experimentally rule out that the Vif G185E substitution indeed did not impact Vif’s functionality we confirmed it by using an HIV-1 Vif deficient luciferase reporter, which was supplemented with expression vectors coding for either wild type or G185E Vif protein.

At the RNA level, in parallel to an increase in exon 3 recognition, mutating G_I3_-2 also decreased levels of exon 2 containing transcripts as well as *vif* mRNA, demonstrating that recognition of either exon strongly influences the other. Indeed, we have shown recently that excessive splicing of exon 3 and *vpr* mRNA processing concomitantly resulted in considerable decrease of exon 2 and *vif* mRNA splicing, indicating an apparently mutually exclusive exon selection of exon 2 and exon 3 [33]. In this work we demonstrated that this competition, which is regulated by G_I3_-2, determines the ability to evade A3G-mediated antiviral effects due to *vif* expression. Hence, an insufficient level of Vif is unable to maintain viral replication due to insufficient A3G-counteraction.

All HIV-1 intron-containing mRNAs that harbor translational start codons in their introns immediately downstream of their leader exon (avoiding translational inhibitory AUGs) depend on the recognition of the leader exons’ 3′ss. However, their corresponding 5′ss must be rendered splicing incompetent in order to include the start codons into the nascent transcript. For instance, the intron-containing *env* mRNAs, which belong to the class of HIV-1 4 kb mRNAs, are formed by using a splice acceptor that is derived from either one of the 3′ss central cluster (A4c, a, b and A5), and splicing repression at D4. Hereby, U1 bound to D4 and U2 snRNPs bound to 3′ss A4cab or A5 pair with each other via cross-exon interactions [35] and facilitate exon definition [59,60]. In addition, these interactions are supported by the strong guanosine-adenosine-rich enhancer GAR ESE, which is localized immediately downstream of 3′ss A5 [35,61]. Importantly, the binding of a splicing incompetent U1 snRNA was sufficient to promote exon definition and 3′ss activation indicating that exon definition but not splicing at D4 is crucial to activate upstream splice acceptor usage in order to gain *env*/*vpu* mRNAs [35]. In a similar way, *vif* and *vpr* mRNAs seem to rely on comparable functional cross-exon interactions, in these cases between splice sites A1 and D2 (exon 2) as well as A2 and D3 (exon 3), respectively, which determined the splicing efficiency of *vif* and *vpr* mRNA. In agreement with the formation of *env*/*vpu* mRNAs, exon 3 inclusion and *vpr* mRNA expression can be modulated by up and down mutations of 5′ss D3 as well as by co-transfection of modified U1 snRNAs with perfect complementarity to the 5′ss D3 [33,37]. Hereby, binding of U1 snRNP to a non-functional 5′ss was shown to be already sufficient to enhance splicing at the upstream 3′ss A2 indicating that *vpr* encoding mRNAs are dependent on the relative occurrence of U1-bound, but splicing-repressed 5′ss [33]. Correspondingly, the co-expression of a U1 snRNA that was fully complementary to a splicing deficient HIV-1 D2 mutant was sufficient to maintain *vif* mRNA formation [32]. Since both D3 up [33] as well as G_I3_-2 mutations increased exon 3 inclusion as well as *vpr* formation, it seems plausible that G_I3_-2 might play a role in the inhibition of U1 snRNP recruitment to D3.

So far *vif* mRNA formation has been known to be maintained by the two SRSF1 dependent heptameric exonic splicing enhancers ESEM1 and ESEM2 [40], the SRSF4 dependent ESE Vif [32], as well by the intronic G rich silencer elements G4 overlapping with the intronic nucleotides of 5′ss D2 and thus likely competing with U1 snRNP binding [32]. In addition, we recently identified the intronic G run G_I2_-1, which impairs usage of the HIV-1 alternative 5′ss D2b as well as exon definition of exon 2b, and thus inhibits splicing at 3′ss A1 [34]. Here, we show that *vif* mRNA is not only regulated by exon 2 and exon 2b associated SREs [32,34,40], but in addition is also controlled by the balanced exon recognition and splicing of exon 2 and exon 3. In contrast to G_I2_-1, G_I3_-2 is entitled as a “deep intronic” splicing regulatory element. The mechanism, although not yet known, seems likely to be different to that described for G_I2_-1. Whereas any splicing regulatory element immediately upstream or downstream of a 5′ss exerts a clear positional splicing regulatory effect [62] this is unpredictable for deeper intronic ones. Thus, the intronic G runs, G_I3_-1 and G_I3_-2, extend the repertoire of SREs, acting on *vif* and *vpr* mRNA splicing regulation.

Conserved non-coding sequences often harbor *cis*-regulatory elements that can vary in their sequence. However, since G_I3_-1 and G_I3_-2 are localized in both *vif* and *vpr* ORFs, there is little room left to maintain proper protein affinity forming a compromise between splicing efficiency on the one hand and protein function on the other hand. Comparing HIV-1 consensus sequences (Los Alamos HIV database) it turned out that G_I3_-2 matches the consensus sequences of HIV-1 strains A2, B and D. In addition, the three consensus sequences of strains J, G and AE were equivalent to the inactivating G_I3_-2 mutation but contained a high affinity hnRNP F/H binding site in position of G_I3_-1 (comparable to G_I3_-1 cmp/G_I3_-2 mut). However, most of the consensus sequences contain both G runs, G_I3_-1 and G_I3_-2, as high affinity binding sites. Thus, removal of only a single G run preserves phenotypic functioning indicating that a single protein binding site irrespective of the exact nucleotide sequence is sufficient to maintain proper splicing. Since viral replication of G_I3_-2 mutant NL4-3 virus was considerably impaired in A3G-expressing, but not in -deficient cells, we propose that at least one functional high affinity binding site for hnRNP F/H and A2/B1, either G_I3_-1 or G_I3_-2, is critical to maintain an optimal Vif to A3G ratio. Further support for this model comes from previous studies showing that siRNA-directed down-regulation of hnRNP A2/B1 proteins within pNL4-3-transfected HEK 293 T cells leads to a viral oversplicing phenotype of exon 3 containing and *vpr* coding mRNAs reminiscent to that seen for the G_I3_-2 mutant [63,64]. In addition, the redundancy of these G runs could represent a viral backup mechanism to easily re-substitute defect binding sites by an exchange of a single nucleotide.

Targeting Vif gene expression represents an attractive therapeutic strategy as it supports infected cells to defend themselves in an APOBEC3-dependent manner. Since viral replication of G_I3_-2 mutant NL4-3 virus was strongly impaired in human primary T-lymphocytes, G runs G_I3_-1 and G_I3_-2 may represent suitable therapeutic targets. Therefore we tested the effect of an LNA specifically designed to target G_I3_-2 on viral particle production. Surprisingly, we found that targeting G_I3_-2 had an even more dramatic effect on the viral particle production than inactivating G_I3_-2 upon mutation. This apparent stronger effect might to some extend result from an yet unknown degradation mechanism of the viral target RNAs. Since we used mixmer LNAs (combination of LNA and DNA residues) it is highly unlikely that they recruit RNaseH, which has been shown to need at least a gap of 6 neighbouring deoxynucleotides for noteworthy RNase H activity [65,66]. Although the mechanism of action is not yet fully understood, viral particle production seems to be specific as the control mismatch LNA did not cause any harm.

Since sublethal levels are proposed to contribute to viral genetic diversity, suboptimal Vif inactivation might give rise to the emergence of viral quasi-species and drug resistant HIV-1 strains [1,67,68]. Hence, there is a need for multiple therapeutic approaches to inactivate Vif in parallel. Potentially, this can be achieved by masking numerous SREs that facilitate *vif* expression. Furthermore, this strategy could minimize the risk of second site mutations that may potentially substitute therapeutically induced aberrant splicing. Moreover, it will be interesting to analyze the effect of G_I3_-2-mutation derived increase of Vpr protein levels, which are important for HIV-1 replication in macrophages.

## Conclusions

Our data suggest that the intronic G runs G_I3_-1 and G_I3_-2, which are functionally conserved in most HIV-1 strains, are critical for efficient viral replication in A3G-expressing but not in A3G-deficient T cell lines. Hereby, inactivation of G_I3_-2 results in increased levels of both mRNA and protein levels of Vpr, but concomitantly in decreased amounts of Vif mRNA and protein levels. G_I3_-2, which is bound by hnRNP F/H and A2/B1 proteins, plays a major role in the apparent mutually exclusive exon selection of *vif* and *vpr* leader exon selection. Furthermore, mutating G_I3_-2 decreased viral mRNA levels, altered the ratio of unspliced 9 kb mRNA and thus reduced viral production. Since competition between these exons determines the ability to evade A3G-mediated antiviral effects due to *vif* expression, we propose that G_I3_-2 is critical for viral replication in non-permissive cells due to an optimal Vif-to-A3G ratio as well as for maintenance of efficient virus production.

## Methods

### Plasmids

Proviral DNA pNL4-3 G_I3_-2 mut was generated by replacing the AflII/NarI fragment of pNL4-3 [GenBank: M19921] [69] by the PCR-amplicon obtained by using primer pair #2339/#3896 (for all sequences see Table 1). Proviral plasmid pNL4-3 G_I3_-3 mut was generated by substitution of the EcoRI/NdeI fragment of pNL4-3 with a PCR product containing equal restriction sites by using primer pair #2330/#3897. The respective PCR products for pNL4-3 G_I3_-1 cmp (#4355/#4718) and pNL4-3 G_I3_-1 cmp/G_I3_-2 mut (#4355/#4720) containing PflMI and XcmI restriction sites were cloned into pNL4-3 by substitution of the PflMI/XcmI fragment. Due to the overlapping *vif* and *vpr* open reading frames (ORFs), mutations resulted in single amino acid substitutions (K181R G_I3_-1 cmp; G185E G_I3_-2 mut) within the Vif protein (Figure 8D). pXGH5 [70] was co-transfected to monitor transfection efficiency in quantitative and semi-quantitative RT-PCR analyses. pcDNA3.1 Vif and pcDNA Vif-V5 plasmids [71] were used to introduce point mutation G185E by site directed mutagenesis using PCR primers (vifmut-forward/vifmut-reverse). PCR products were treated for 1 h at 37°C with 10 units of DpnI restriction enzyme to digest the parental methylated plasmids and transformed into *E. coli*. All PCR-amplified sequences of the plasmids were validated by DNA-sequencing.

**Table 1:** **DNA oligonucleotides used in this work**

**Primer**	**Primer sequence**
#0640	5′- CAATACTACT TCTTGTGGGT TGG
#1544	5′- CTTGAAAGCG AAAGTAAAGC
#2330	5′- TCTGGATCCA CCACCACCAC CGTAGAT
#2339	5′- TGGGAGCTCT CTGGCTAACT AGGGAACCCACTGCTTAAGC
#3153	5′- CCACTCCTCC ACCTTTGAC
#3154	5′- ACCCTGTTGC TGTAGCCA
#3387	5′- TTGCTCAATG CCACAGCCAT
#3388	5′- TTTGACCACT TGCCACCCAT
#3389	5′- TTCTTCAGAG CAGACCAGAG C
#3390	5′- GCTGCCAAAG AGTGATCTGA
#3391	5′- TCTATCAAAG CAACCCACCTC
#3392	5′- CGTCCCAGAT AAGTGCTAAGG
#3395	5′- GGCGACTGGG ACAGCA
#3396	5′- CCTGTCTACT TGCCACAC
#3397	5′- CGGCGACTGA ATCTGCTAT
#3398	5′- CCTAACACTA GGCAAAGGTG
#3631	5′- CGGCGACTGA ATTGGGTGT
#3632	5′- TGGATGCTTC CAGGGCTC
#3633	5′- CGACACCCAA TTCTTGTTAT GTC
#3636	5′- CCGCTTCTTC CTTGTTATGT C
#3896	5′- TTCACTCTTA AGTTCCTCTA AAAGCTCTAG TGTCCATTCA TTGTATGGCT CTCTCTGTGG CCCTTGGTCT TCTG
#3897	5′- GTTGCAGAAT TCTTATTATG GCTTCCACTC CTGCCCAAGT ATCGCCGTAA GTTTCATAGA TATGTTGTCC TAAGTTATG
#4324	5′- TAATACGACT CACTATAGG
#4355	5′- TTCATCGAAT TCAGTGCCAA GAAGAAAAGC AAAGATCA
#4614	5′- TTCATTGTAT GGCTCCCTCT GTGGCCCTTG ACATGGGTGA TCCTCATGTC CTATAGTGAG TCGTATTA
#4615	5′- TTCATTGTAT GGCTCTCTCT GTGGCCCTTG ACATGGGTGA TCCTCATGTC CTATAGTGAG TCGTATTA
#4718	5′- TAGTGTCCAT TCATTGTATG GCTCCCTCTG TGGCCCCTGG T
#4720	5′- TAGTGTCCAT TCATTGTATG GCTCTCTCTG TGGCCCCTGG T
#4843	5′- CCGCTTCTTC CTTTCCAGAG G
#4849	5′- CCTCTGGAAA GAATTGGGT
vifmut-forward	5′- AGGGCCACAG AGAGAGCCAT ACAATG
vifmut-reverse	5′- CATTGTATGG CTCTCTCTGT GGCCCT

### Oligonucleotides

All DNA oligonucleotides (Table 1) were obtained from Metabion (Germany), those used for real time PCR analysis were HPLC purified. RNase-Free HPLC purified LNAs (G_I3_-2: TATGGCTCCCTCTGTG; G_I3_-2 mismatch control: TTTGGCTCACTCCGTG) were purchased from Exiqon (Denmark).

### Cell culture, transfection conditions and preparation of virus stocks

HEK 293 T and HeLa cells were maintained in Dulbecco’s high glucose modified Eagle’s medium (Invitrogen) supplemented with 10% (v/v) heat-inactivated fetal calf serum (FCS) and 50 μg/ml of penicillin and streptomycin (P/S) each (Invitrogen). Transient transfection experiments were performed in six-well plates (2.5 × 10^5^ cells per well) using TransIT®-LT1 transfection reagent (Mirus Bio LLC) according to the manufacturer’s instructions. For LNA co-transfection experiments, 2.5 × 10^5^ HeLa cells per well (six-well plate) were cultured in Opti-MEM reduced serum medium (Invitrogen) with 5% FCS. The next day, medium was replaced with Opti-MEM reduced serum medium without FCS. For LNA transfection 4 μl of Lipofectamine 2000 (Invitrogen) was added to 250 μl Opti-MEM reduced serum medium. Separately, proviral plasmid pNL4-3 (0.7 μg), plasmid pXGH5 (0.7 μg) and the respective LNAs (80 nM) were added to 250 μl Opti-MEM reduced serum medium. After 5 min the LNA/DNA mixtures were added to the Lipofectamine 2000 containing medium, incubated for 20 min and subsequently added to the cells. After 4 hours, medium was removed and cells were washed twice with PBS and cultured with Opti-MEM reduced serum medium with 5% FCS for 24 hours.

For preparation of virus stocks 6.5 x 10^6^ HEK 293 T cells were cultured in T175 flasks that were previously coated with 0.1% gelatine solution. Cells were transiently transfected with 9 μg of pNL4-3 or mutant proviral DNA using polyethylenimine (Sigma-Aldrich). Following overnight incubation, cells were supplemented with fresh IMDM cell culture medium containing 10% FCS and 1% P/S. 48 hours post transfection, virus containing supernatant was purified by centrifugation, aliquoted and stored at −80°C. Transfection efficiency was monitored by using pNL4-3 GFP [72].

CEM-A and CEM-SS cells were maintained in RPMI 1640 medium (Invitrogen) supplemented with 10% FCS and P/S (50 μg/ml each, Invitrogen). Peripheral blood mononuclear cells (PBMCs) were isolated from 15 ml whole blood from two healthy donors by ficoll gradient centrifugation. PBMCs were maintained in RPMI 1640 GlutaMax medium containing 10% FCS and 1% P/S and activated with phytohemagglutinin PHA (5 μg/ml). 48 hours post isolation cells were treated with IL-2 (30 mg/ml).

### RNA-isolation, quantitative and semi-quantitative RT-PCR

Total RNA was isolated by using acid guanidinium thiocyanate-phenol-chloroform as described previously [73]. RNA concentration and quality was analyzed by photometric measurement using Nano-Drop 1000 spectrophotometer, ND-1000 version 3.7.0 (Thermo Scientific). Reverse transcription of 5 μg of total RNA was performed as described previously [34]. For quantitative and qualitative analysis of HIV-1 mRNAs the indicated primers (Table 1) were used to amplify the cDNA-template. As a loading control, a separate PCR detecting GAPDH was performed with primers #3153 and #3154. PCR products were separated on non-denaturing polyacrylamide gels (10%), stained with ethidium bromide and visualized with the Intas Gel iX Darkbox II (Intas, Germany). Quantitative RT-PCR analysis was performed by using Precision 2× real-time PCR MasterMix with SYBR green (Primerdesign, UK) using LightCycler 1.5 (Roche). Primers used for qualitative and quantitative RT-PCR are listed in Table 1.

### Protein isolation and Western blotting

For protein isolation cells were lysed using RIPA lysis buffer (25 mM Tris HCl [pH 7.6], 150 mM NaCl, 1% NP-40, 1% sodium deoxycholate, 0.1% SDS, protease inhibitor cocktail [Roche]). Subsequently, the lysates were subjected to SDS-PAGE under denaturating conditions [74] in 8-12% polyacrylamide gels (Rotiphorese Gel 30, Roth) as described before [34]. The following primary antibodies were used for immunoblot analysis: Sheep antibody against HIV-1 p24 CA from Aalto (Ireland); mouse monoclonal antibodies specific for HIV-1 Vif (ab66643) and hnRNP F + H proteins (ab10689) from Abcam (United Kingdom); hnRNPA2/B1 (DP3B3): sc-32316 from Santa Cruz; rabbit anti-HIV-1-Vpr (51143-1-AP) polyclonal antibody from Proteintech Group (United Kingdom); rabbit polyclonal antibody against MS2 (TC-7004) from Tetracore (Rockwill, USA); mouse anti β-actin monoclonal antibody (A5316) from Sigma-Aldrich. The following horseradish peroxidase (HRP) conjugated secondary antibodies were used: anti-rabbit HRP conjugate (A6154) from Sigma-Aldrich; anti-mouse antibody (NA931) from GE Healthcare (Germany), and anti-sheep HRP from Jackson Immunoresearch Laboratories Inc. (West Grove, PA). Blots were visualized by an ECL chemiluminescence detection system (Amersham) and Intas ChemoCam imager (Intas, Germany).

For analysis of expression of viral structural proteins, Vif and A3G transfected cells were harvested, washed with PBS and lysed using RIPA assay buffer for 20 min on ice [25 mM Tris (pH 8.0), 137 mM NaCl, 1% glycerol, 0.1% SDS, 0.5% Na-deoxycholate, 1% Nonidet P-40, 2 mM EDTA, and complete protease inhibitor mixture (Roche)]. Soluble lysates were clarified by centrifugation and subjected to SDS-PAGE followed by transfer to a PVDF membrane (Millipore). Viral particles were concentrated by ultracentrifugation over 20% sucrose cushion (in PBS) in ultra-clear centrifuge tubes (13 × 51 mm, Beckman Coulter) and centrifuged at 37,000 rpm for 2 h at 4°C in an MLS-50 rotor (Beckman Coulter). Pelleted particles were lysed by RIPA assay buffer and directly subjected to immunoblotting. Membranes were probed with mouse anti-HA antibody to detect A3G (1:10^4^ dilution; Covance, Munich, Germany), mouse anti-Vif antibody (1:10^3^) [75] p24 was detected using mouse p24/p27 monoclonal antibody AG3.0 (1:250) [76]. Mouse anti-VSV-G (1:2× 10^4^ dilutions; Sigma-Aldrich), anti-mouse horseradish peroxidase (1:10^4^ dilution; GE Healthcare, Munich, Germany). Alpha-tubulin was detected using an anti-tubulin antibody (1:10^4^ dilution; Sigma-Aldrich). Signals were visualized by ECL prime reagent (GE Healthcare).

### Northern blotting

For Northern blotting of HIV-1 mRNAs 3 μg of total RNA were separated on denaturing 1% agarose gel and capillary blotted onto positively charged nylon membrane and hybridized with an digoxigenin (DIG)-labeled HIV-1 exon 7 PCR-amplicon (#3387/#3388) as previously described [34].

### Measurement of HIV-1 replication kinetics

Virus containing supernatants, which were generated by transient transfection of HEK 293 T cells (see above), were assayed for p24-CA via p24-CA ELISA or alternatively for TCID_50_ as determined by calculation of X-Gal stained TZM-bl cells. 4 × 10^5^ CEM-SS or CEM-A cells were infected with 1.6 ng of p24-CA of WT and mutant viruses in serum-free RPMI medium at 37°C for 6 hrs. Infected cells were washed in PBS (Invitrogen) and resuspended in RPMI media (Invitrogen) containing 10% FCS and 1% P/S (Invitrogen). Aliquots of cell-free media were harvested at intervals and subjected to p24-CA ELISA (see below). 8 × 10^5^ PBMCs were infected with the indicated MOI as determined by TCID_50_ calculation.

### p24-CA ELISA

For HIV-1 p24-CA quantification using twin-site sandwich ELISA [77,78] Nunc-Immuno 96 MicroWell solid plates (Nunc) were coated with anti p24 polyclonal antibody (7.5 μg/ml of D7320, Aalto) in bicarbonate coating buffer (100 mM NaHCO_3_ pH 8.5) and incubated overnight at room temperature. Subsequently, the plates were washed with TBS (144 mM NaCl, 25 mM Tris pH 7.5). HIV in the cell culture supernatant was inactivated by adding Empigen zwitterionic detergent (Sigma, 45165) followed by incubation at 56°C for 30 min. After p24 capturing and subsequent TBS-washing, sample specific p24 was quantified by using an alkaline phosphatase-conjugated anti-p24 monoclonal antibody raised against conserved regions of p24 (BC 1071 AP, Aalto) using the AMPAK detection system, (K6200, Oxoid (Ely) Ltd). For a p24 calibration curve, recombinant p24 was treated as described above.

### Infectivity assay

Transfections of HEK 293 T cells were performed using Lipofectamine LTX reagent (Invitrogen, Karlsruhe, Germany). HIV-1 luc reporter vectors were produced in the presence and absence of A3G, wild type (wt) Vif, wt Vif-V5, Vif G185E and Vif-V5 G185E or empty vector (pcDNA3.1) by transient transfection of 600 ng of pMDLg/pRRE, 250 ng of pRSV-Rev, 150 ng of pMD.G (VSV-G) and 600 ng of reporter vector pSIN.PPT.CMV.Luc.IRES-GFP. The HIV-1 packaging plasmid pMDLg/pRRE encodes *gag-pol* and the pRSV-Rev HIV-1 *rev* [79]. The HIV-1 derived vector pSIN.PPT.CMV.Luc.IRES.GFP expresses the firefly luciferase and GFP. The luciferase cDNA (luc 3) was cloned into NheI and BamHI restriction sites of pSIN.PPT.CMVmcsIRES.GFP [80], a gift of Neeltje Kootsta. A3G and Vif expression plasmids were kept in a ratio of 1:1 (600 ng each). 48 h post transfection, viral particles containing supernatants were collected and stored at −80°C until they were used. RT activity was quantified using the Lenti-RT Activity Assay (Cavidi Tech, Uppsala, Sweden).

To determine virus rescuing activity of wt Vif and Vif G185E (and the V5 tagged version) HEK 293 T cells were transduced in the presence or absence of A3G with normalized amounts of viral like particles (determined by RT activity). Two days after transduction, intracellular luciferase activity was quantified using Steady Lite HTS (Perkin-Elmer). Data were presented as the average of actual luciferase activity per ten seconds of the quadruplicate (with mean and standard deviations). Statistically significant differences between two groups were analyzed using unpaired Student’s *t* test in GraphPad Prism, version 5 (GraphPad Software, San Diego, CA). A minimum *P* value of 0.05 was considered statistically significant.

### HIV-sequence alignments and sequence logos

HIV-1 sequences were downloaded from the Los Alamos HIV-1 Sequence Compendium 2012 (http://www.hiv.lanl.gov/). The subtype sequences were analysed with the RIP 3.0 software (http://www.hiv.lanl.gov/content/sequence/RIP/RIP.html). Sequence logos were generated by using R Statistical Computing (http://www.r-project.org) and R package seqLogo version 1.28.0 [81].

### RNA pull-down

Pre-annealed DNA oligonucleotides containing G_I3_-2 wt and mutant sequences as well as a single copy of the MS2 binding site and T7 sequences (Table 1) were subjected to *in vitro* transcription using RiboMAX™ Large Scale RNA Production Systems (Promega) according to the manufactures instructions (T7 Primer: #4324; G_I3_-2 wt: #4614; G_I3_-2 mut: #4615). Following a phenol-chloroform extraction, the RNAs were covalently immobilized on adipic acid dihydrazide-agarose beads (Sigma) and incubated in 60% HeLa cell nuclear extract (Cilbiotech) in buffer D (20 mM HEPES-KOH [pH 7.9], 5% [vol/vol] glycerol, 0.1 M KCl, 0.2 mM EDTA, 0.5 mM dithiothreitol). Recombinant MS2 protein (1 μg) was added to compare the input of each sample. Unspecific bound proteins were removed by repetitive washing with buffer D containing 4 mM magnesium chloride. The associated proteins were eluted by heating at 95°C for 10 min, separated via SDS-PAGE (16%) and subjected to immunoblot analysis. For interference with protein:RNA interaction, wt RNA substrate was pre-incubated with the G_I3_-2 LNA in a ratio of either 1:5 or 1:1 relative to the amount of RNA substrate (1000 pmol).

## Additional file

Additional file 1: Figure S1. Binding sites of RT-PCR primers used in this work. Schematic illustration of the positions of all NL4-3 related PCR primers used in quantitative and qualitative RT-PCR assays. Locations of 5′ and 3′ splice sites (ss), exons and introns are indicated. Vif and Vpr exons are highlighted in red and blue. The positions of relevant PCR forward and reverse primers are indicated by black triangles. Primer pairs were used as follows: unspliced 9 kb mRNAs (#3389/#3390), intronless 2 kb mRNAs (#3391/#3392), exon 2 containing viral mRNAs (#3395/#4843), exon 3 containing viral mRNAs (#3397/#3636), *vif* mRNA (#3395/#3396), *vpr* mRNA (#3397/#3398), *tat1* mRNA (#3631/#3632), *tat2* mRNA (#3395/#4849), *tat3* mRNA (#3397/#3632), and all viral mRNAs containing exon 7 (#3387/#3388).

## Abbreviations

SRE: Splicing regulatory element
hnRNP: Heterogeneous ribonucleoprotein particle
HIV: Human immunodeficiency virus
SRSF: SR splicing factor
APOBEC: Apolipoprotein B mRNA-editing enzyme, catalytic polypeptide-like
ORF: Open reading frame
PBMC: Peripheral blood mononuclear cell
ELISA: Enzyme-linked immunosorbent assay
LNA: Locked nucleic acid
snRNP: Small nuclear ribonucleic particle
aa: Amino acid
HBS: HBond score
