# Supplementary figures and images for: A functional conserved intronic G run in HIV-1 intron 3 is critical to counteract APOBEC3G-mediated host restriction
Source: Retrovirology. 2014 Aug 29;11:72. doi: 10.1186/s12977-014-0072-1 (PMC4163160; doi:10.1186/s12977-014-0072-1)

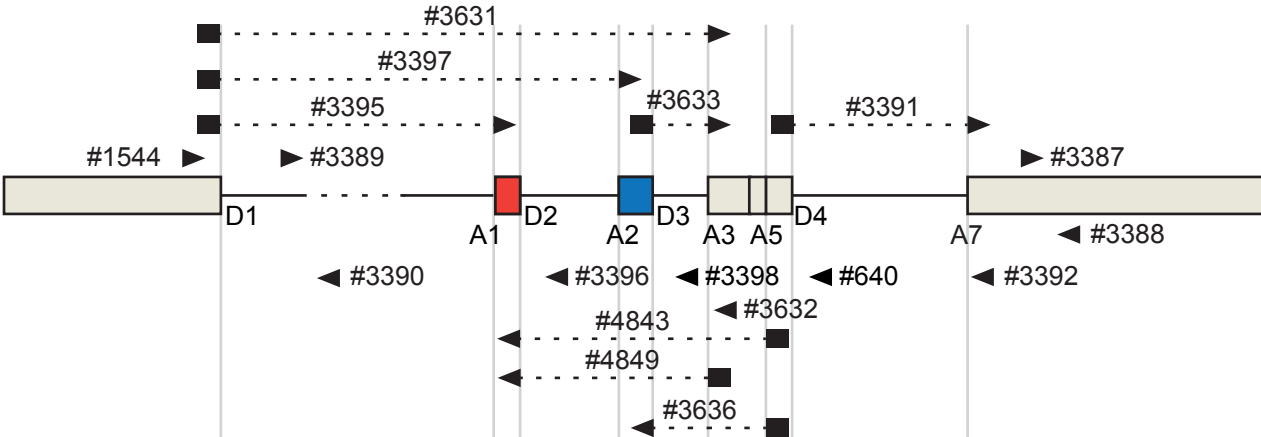

Supplement: Additional file 1: Figure S1. — Binding sites of RT-PCR primers used in this work. Schematic illustration of the positions of all NL4-3 related PCR primers used in quantitative and qualitative RT-PCR assays. Locations of 5′ and 3′ splice sites (ss), exons and introns are indicated. Vif and Vpr exons are highlighted in red and blue. The positions of relevant PCR forward and reverse primers are indicated by black triangles. Primer pairs were used as follows: unspliced 9 kb mRNAs (#3389/#3390), intronless 2 kb mRNAs (#3391/#3392), exon 2 containing viral mRNAs (#3395/#4843), exon 3 containing viral mRNAs (#3397/#3636), vif mRNA (#3395/#3396), vpr mRNA (#3397/#3398), tat1 mRNA (#3631/#3632), tat2 mRNA (#3395/#4849), tat3 mRNA (#3397/#3632), and all viral mRNAs containing exon 7 (#3387/#3388). [file 12977_2014_72_MOESM1_ESM.pdf]
